# Antiviral Activities of Mulberry (*Morus alba*) Juice and Seed against Influenza Viruses

**DOI:** 10.1155/2018/2606583

**Published:** 2018-11-01

**Authors:** Hyojin Kim, Mi Sook Chung

**Affiliations:** Department of Food and Nutrition, Duksung Women's University, Seoul 01369, Republic of Korea

## Abstract

Antiviral activities of* Morus alba* (MA) juice and seed were examined using time-of-addition plaque assays against influenza viruses, A/Brisbane/59/2007 (H1N1) (BR59), pandemic A/Korea/01/2009(H1N1) (KR01), A/Brisbane/10/2007(H3N2) (BR10), and B/Florida/4/2006 (FL04). MA juice (MAJ) showed much higher antiviral activity than MA seed (MAS). In the pre- and cotreatment of virus, MAJ showed antiviral effects against BR59, KR01, and FL04 in a dose-dependent manner. In particular, MAJ at 4% concentration exhibited 1.3 log inhibition in the pre- and cotreatment of the virus against FL04, a type B virus. However, little or weak inhibition was observed in the posttreatment of MAJ. GSH levels in the virus-infected cells were also examined. The decreased levels by the viral infection were restored significantly by the addition of MAJ. MAJ also exhibited significant DPPH radical scavenging and ferric ion-reducing activities in a dose-dependent manner. Cyanidin-3-rutinoside, the most abundant polyphenol compound of MAJ identified by LC-MS in this study, showed weak inhibitory effects against FL04 in the pretreatment, whereas gallic acid, a minor compound of MAJ, revealed significant antiviral effect. These results suggest that MAJ can be developed as a novel plant-derived antiviral against influenza viruses.

## 1. Introduction

Influenza viruses cause acute respiratory infection responsible for seasonal epidemics and pandemics, thereby imposing a huge toll on both human health and the economy worldwide [1, 2]. Influenza viruses that belong to the* Orthomyxoviridae* family are classified as types A, B, C, and recently identified D [3, 4]. Human influenza A and B viruses cause seasonal epidemics of respiratory diseases almost every winter. Type A influenza viruses are further divided into subtypes based on two proteins on the surface of the virus: the hemagglutinin (HA) and the neuraminidase (NA). There are 18 HA (H1-18) and 11 NA subtypes (N1-11) [5]. Type A and B influenza viruses contain 8 single-stranded viral RNA gene segments, which encode transcripts for 10 essential viral proteins, as well as several strain-dependent accessory proteins [4]. However, influenza type C and D viruses only possess seven viral RNA gene segments [3]. At present, two classes of antiviral drugs, the M2 ion channel inhibitors (amantadine and rimantadine) and NA inhibitors (oseltamivir and zanamivir), have proven effective in preventing influenza viral infection [6, 7]. Adamantine inhibits the ability of the viral ion channel protein M2 to exchange H^+^ in order to lower the pH inside the virus during viral fusion and uncoating. Oseltamivir inhibits NA protein, blocking the release of newly formed virions from infected host cells. These antiviral compounds are efficacious against influenza virus strains, but an alarming proportion of the circulating seasonal influenza A virus has become resistant to both oseltamivir and amantadine due to mutations in the viral amino acid sequence [8, 9]. A recent virus strain, A/Hong Kong/2652/2006-like viruses (H1N1), showed double resistance to amantadine and oseltamivir [8]. Therefore, new broad-spectrum therapeutic approaches and alternative strategies are urgently needed for the control of influenza.

Various plant extracts with anti-influenza activity have been recently identified. These include cocoa, guava tea, green tea by-products,* Pelargonium sidoides* root,* Plumbago indica* root,* Alpinia katsumadai* seed,* Rubus coreanus* seed, and* Jatropha multifida* Linn. stem [10–17]. In addition, anti-influenza constituents, including polyphenols, of plant were identified: quercetin-3-gallate, cardiotonic glycosides from* Adenium obesum*, theaflavin derivatives, catechins, resveratrol, chlorogenic acid, and dendrobine [18–24].* Morus alba* (MA), which belongs to the* Moraceae* plant, is a species of mulberry containing 1-deoxynojirimycin with antiviral effects against hepatitis B and C viruses [25]. MA leaf extract possesses significant antibacterial effects on* Aggregatibacter actinomycetemcomitans*,* Porphyromonas gingivalis*, and* Tannerella forsythia *[26]. Cyanidin-3-glucoside, a major phenolic compound of mulberry fruit, shows neuroprotective effects against oxygen-glucose deprivation [27]. MA seeds (MAS) are mostly by-products of wine and juice production and contain large quantities of phenolic compounds [28]. MA juice (MAJ) is rich in polyphenols with biological activities that may impact positively on human health. The MAJ and MAS showed antiviral effects against foodborne viral surrogates [28, 29]. Nevertheless, antiviral activities of MAJ and MAS against influenza viruses have not been explored previously.

Influenza virus infection was recently found to activate reactive oxygen species (ROS) [30].* Humulus lupulus* extract and some antioxidant molecules, including polyphenols, that are able to modulate the intracellular redox balance, showed anti-influenza activity [31–34]. In the present study, the antiviral effects of MAJ and MAS were examined against influenza virus strains, A/Brisbane/59/2007(H1N1) (BR59), pandemic A/Korea/01/2009(H1N1) (KR01), A/Brisbane/10/2007(H3N2) (BR10), and B/Florida/4/2006 (FL04). MAJ with significant antiviral effects against BR59, KR01, and FL04 in a dose-dependent manner was further examined for its antioxidant activity.

## 2. Materials and Methods

### 2.1. Chemicals and Materials

The chemicals, including phosphate buffered saline (PBS), 3-(4,5-dimethylthiozol-2-yl)-3,5-dipheryl tetrazolium bromide (MTT), fetal bovine serum (FBS), glutathione (GSH), glutathione reductase (GR), 5,5′-dithiobis(2-nitrobenzoic acid) (DTNB), nicotinamide adenine dinucleotide phosphate (NADPH), 1,1-diphenyl-2-picrylhydrazyl (DPPH), 2,4,6-tris(2-pyridyl)-1,3,5-triazine (TPTZ), and ascorbic acid, were purchased from Sigma-Aldrich Co. (St. Louis, MO, USA). Dulbecco's modified Eagle's medium (DMEM) and penicillin-streptomycin (PS) were purchased from Gibco-Invitrogen (Carlsbad, CA, USA). Fruits of MA were purchased from Sununsan Nonghyup, Jeonnam, Korea. The MAS was obtained as a gift from Gochang Whangto Bokbunja, Jeonnam, Korea. Mulberry fruits under investigation were identified by staff members of Natural Academy of Agricultural Science, Rural Development Administration, Korea.

### 2.2. Viruses and Cell Lines

The influenza strains, BR59, KR01, BR10, and FL04, were obtained from the Korea Centers for Disease Control and Prevention. Madin-Darby canine kidney (MDCK) cell line was purchased from America Type Culture Collection (ATCC; Manassas, VA, USA).

### 2.3. Preparation of Juice and Seed of MA

MAJ was prepared as described previously [29]. Briefly, squeezed juice of MA was filtered through cheesecloth. The filtrate was incubated in a shaking water bath at 63°C for 30 min and centrifuged at 14,000 g for 30 min at 4°C. The supernatant was sterilized by filtration through a 0.20 *μ*m filter (Advantec, Toyo Roshi Kaisha Ltd. Japan) and sterilized juice (MAJ) was stored at −78°C until use. The MAJ was diluted to the indicated concentrations in DMEM.

Freeze-dried seeds were ground into a fine powder with an electric grinder and extracted in 70% ethanol using an ultrasonic bath (40 kHz, Powersonic 420, Hwashin Instrument Co. Ltd., Seoul, Korea) for 20 min at 20°C and then centrifuged (9,500 g, 60 min, 4°C) [28]. After centrifugation, the supernatants were collected, concentrated by rotary evaporator at 40°C, and lyophilized. Seed extract powder (MAS) was then dissolved in PBS (pH 7.0), sterilized by filtration through 0.20* μ*m filter, and diluted aseptically to the indicated concentration in DMEM.

### 2.4. Cytotoxicity Assay

MDCK cells were used at a density of 1.5 × 10^4^ viable cells per well. Cells were seeded in 96-well tissue culture plates in DMEM containing 10% heat-inactivated FBS and 1% PS. Inhibitor (MAJ or MAS) in DMEM containing 10% FBS was added and incubated for 12 h at 37°C and 5% CO_2_. After the culture medium was removed, 10* μ*L of MTT was added to each well, followed by incubation at 37°C for 4 h. After removal of the solution, 100* μ*L of dimethyl sulfoxide (DMSO, Sigma-Aldrich) was added and incubated for 10 min. Absorbance at 570 nm was measured in a microplate reader (SpectraMax M2, Molecular Devices Corp. USA). The percentage of cell viability after treatment with inhibitor was calculated as follows: % cell viability = (Abs_treatment_ /Abs_control_) ×100.

### 2.5. Plaque Reduction Assays

Antiviral activity was evaluated by plaque reduction assays using time-of-addition experiments as described previously [16]. Pretreatment of virus with inhibitor was conducted by incubating inhibitor (MAJ, MAS, or polyphenol compound) and the virus (6-7 log_10_ PFU/mL) (1:9 ratio) at room temperature for 1 h. Viral suspensions were serially diluted 10-fold in DMEM medium and inoculated onto confluent cell monolayers at 37°C in 5% CO_2_ for 1 h. After viruses were adsorbed, inocula were aspirated, after which 1 mL of 1.5% low melting point agarose overlay prepared in culture medium containing 2* μ*g/mL of trypsin was added to each well. After incubating plates at 37°C in 5% CO_2_ for 48-72 h, the cell monolayer was fixed with 4% formaldehyde for 1 h. Then, agarose overlay was removed and the cell layer was stained with 0.5% crystal violet. Plaques were counted and sterilized distilled water was used as the untreated control. Data were represented as plaque-forming unit (PFU) reduction (log_10_PFU/mL).

For the cotreatment, the same experimental procedures as the pretreatment of virus were followed, except that confluent cell monolayers were infected with 180* μ*L of virus suspensions (6-7 log_10_ PFU/mL) and simultaneously mixed with 20* μ*L of inhibitor at 37°C in 5% CO_2_ for 1 h. For the posttreatment, after 180* μ*L of virus (6-7 log_10_ PFU/mL) adsorption to cells, inocula were aspirated and cells were incubated with inhibitor for 1 h. After incubation, the inhibitor was aspirated and 1 mL of 1.5% low melting point agarose overlay prepared in culture medium containing 2* μ*g/mL of trypsin was added to each well. The remainder of the procedure was the same as the pretreatment of virus.

### 2.6. GSH Assay

GSH level was determined as previously described [35] with minor modifications. Briefly, MDCK cells were seeded at a density of 0.5 × 10^6^ per well in a 24-well plate, and the cells were grown ~90% confluency. Cells were infected with KR01 or FL04, which were treated with 4% MAJ as follows: (1) nontreated influenza virus (IV), (2) virus-infected and cotreatment with MAJ (IV + MAJ), (3) cotreatment with MAJ and incubation 1 h after infection (IV + MAJ + 1 hpi), and (4) cotreatment with MAJ and incubation 24 h after infection (IV + MAJ + 24 hpi). The cell lysates from each treatment were mixed with 120 *μ*L of 5,5′-dithio-bis(2-nitrobenzoic acid) (DTNB) and glutathione reductase (GR) mixture for 30 seconds. After 60 *μ*L of NADPH was added to the cell lysate, absorbance at 412 nm was measured in a microplate reader (SpectraMax M2). The GSH levels were expressed as percentage of nM/10^6^ cells (where levels from control cells were considered 100%).

### 2.7. DPPH Radical Scavenging Activity

DPPH radical scavenging activity was evaluated as described by Shimada et al. [36] with minor modification. The 100 *μ*L of sample was mixed with 100 *μ*L of 0.3 mM DPPH solution. The mixture was shaken vigorously and incubated for 30 min and the absorbance at 515 nm was measured in a microplate reader (SpectraMax M2). Calibration curve was prepared using ascorbic acid at the concentrations of 1-50 *μ*g/mL (r^2^ = 0.999). The percentage of scavenging activity was calculated as follows: % scavenging activity = [(Abs_control_ - Abs_sample_)/Abs_control_] × 100.

### 2.8. Ferric Reducing Antioxidant Power (FRAP) Assay

The antioxidant activity of MAJ was estimated according to the procedure described by Benzie and Strain [37] with some modifications. Briefly, 150* μ*L of FRAP reagent, prepared freshly and warmed at 37°C, was mixed with 50 *μ*L of MAJ; distilled water was used as the reagent blank. The FRAP reagent contained 1 mL of 10 mM TPTZ solution in 40 mM HCl plus 1 mL of 20 mM FeCl_3_·6H_2_O and 10 mL of 0.3 M acetate buffer (pH 3.6). Absorbance at 593 nm was measured in a microplate reader (SpectraMax M2). Aqueous solutions of known Fe (II) concentrations in the range of 15.6-125* μ*M (FeSO_4_·7H_2_O) were used for calibration (r^2^= 0.999). Ascorbic acid was used as antioxidant standard and positive control. The absorbance of the samples was compared to a FeSO_4_ standard curve and the FRAP values were expressed as* μ*M of ferrous equivalent.

### 2.9. Identification of Polyphenol Compounds by LC-MS

Polyphenol compounds of MAJ were quantitatively analyzed using a liquid chromatography mass spectrometer system, LC-MS-8040™ (Shimadzu, Kyoto, Japan) with an electrospray ionization probe and a Nexera UHPLC system equipped with a phenyl-hexyl column (2.1 × 100 mm, 3.5* μ*m; Agilent, Santa Clara, CA, USA). The mobile phase was composed of solvents A (0.1% formic acid) and B (0.1% formic acid in acetonitrile), and the gradient was 95:5 at 1.5 min to 65:35 at 8 min to 0:100 at 10 min, followed by washing and reconditioning of the column. The flow rate was 0.4 mL/min, and the eluent was detected at 280 nm. Standard polyphenol compounds purchased from Sigma-Aldrich Co. were identified as previously reported [38, 39].

### 2.10. Statistical Analysis

All measurements were carried out in triplicate. Data were expressed as the mean ± standard deviation. IBM SPSS Statistics (version 24, IBM Corp, New York, USA) was used for data analysis. Statistical analysis was performed with* t*-test. The significance level was indicated by ^*∗*^*P* < 0.05. For multigroup comparison, the data were analyzed by ANOVA, and the mean values were compared with Tukey's test at the 5% significance level.

## 3. Results

### 3.1. Antiviral Effects of MAJ and MAS

**Figure 1:**
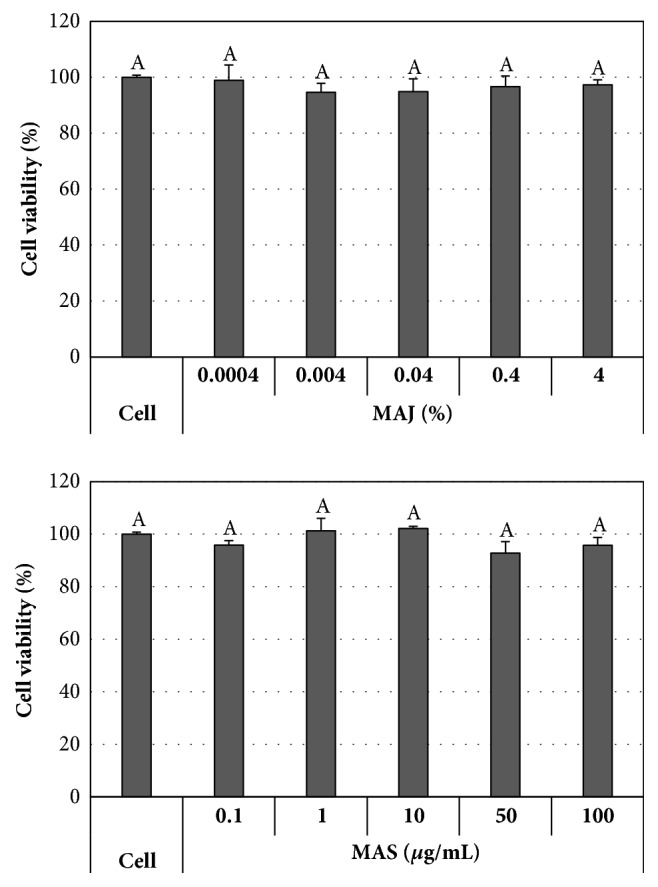
Cytotoxicity of MAJ and MAS. MDCK cells were treated with MAJ or MAS and cytotoxicity was measured by MTT assay. Data were expressed as the mean ± standard deviation from triplicate tests. There was no significant difference in the cell viability of MAJ or MAS in the range of the tested concentrations (*P* < 0.05).

Cytotoxicity studies demonstrated that MAJ and MAS exhibited 95% and 93% cell viability in MDCK cells at concentrations of up to 4% and 100 *μ*g/mL, respectively (Figures 1(a) and 1(b)). There was no significant difference in the cell viability of MAJ or MAS in the range of the tested concentration. Subsequent assays were carried out at concentrations without cytotoxicity. The solvent DMSO had no effect on MDCK cell viability and growth in the range of concentrations used in this study.

**Figure 2:**
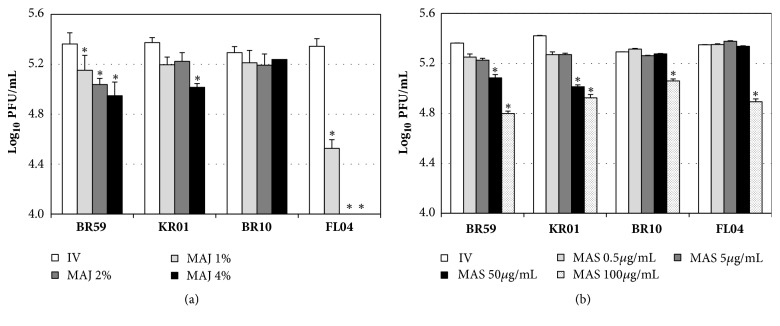
Antiviral activities of MAJ and MAS against BR59, KR01, BR10, and FL04 in pretreatment of virus. Each virus was mixed with (a) MAJ and (b) MAS for 1 h prior to viral infection. After infection, the cells were washed and overlaid with agarose at 37°C for 72 h. Nontreated influenza virus (IV) and oseltamivir (10* μ*M) were used as negative and positive controls, respectively. In the pretreatment of virus, oseltamivir showed no inhibitory effect (data not shown). All measurements were performed in triplicate. Within each treatment, an asterisk denotes a significant decrease in the plaque-forming unit (PFU) (log_10_PFU/mL) to nontreated influenza virus group (*P *< 0.05).

In the pretreatment of virus, MAJ showed inhibitory effects against BR59 (H1N1), KR01 (H1N1), and FL04 (B) in a dose-dependent manner (Figure 2(a)). MAJ at 1% concentration showed 0.2 log inhibition against BR59 and KR01, whereas it exhibited 0.8 log inhibition against FL04, a type B virus, at the same concentration. Notably, it showed higher than 1.3 log inhibition against FL04 at both 2% and 4% concentrations, while it exhibited 0.5 and 0.4 log inhibitions against BR59 and KR01, respectively, at 4%. In contrast, MAJ showed weak inhibition against BR10 (H3N2). In the case of MAS, 0-0.4 log inhibition at 50* μ*g/mL and 0.2-0.6 log inhibition at 100* μ*g/mL were observed against BR59, KR01, BR10, and FL04 (Figure 2(b)).

**Figure 3:**
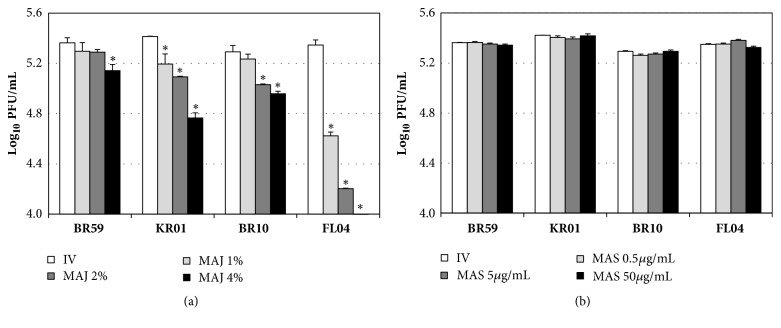
Antiviral activities of MAJ and MAS against BR59, KR01, BR10, and FL04 in cotreatment. Cells were infected with the virus and simultaneously treated with (a) MAJ and (b) MAS for 1 h at 37°C. After infection, the cells were washed and overlaid with agarose at 37°C for 72 h. Nontreated influenza virus (IV) and oseltamivir (10* μ*M) were used as negative and positive controls, respectively. In the cotreatment, oseltamivir showed no inhibitory effect (data not shown). All measurements were performed in triplicate. Within each treatment, an asterisk denotes a significant decrease in the plaque-forming unit (PFU) (log_10_PFU/mL) to nontreated influenza virus group (*P *< 0.05).

In the cotreatment, MAJ showed inhibitory effects against BR59, KR01, BR10, and FL04 in a dose-dependent manner (Figure 3(a)). MAJ showed 0.1-0.2 and 0.1-0.3 log inhibitions at 1% and 2% concentrations, respectively, against BR59, KR01, and BR10. At the concentration of 4%, MAJ showed 0.2-0.6 log inhibition against BR10, BR59, and KR01. Notably, MAJ exhibited strong inhibition reaching 1.1 and 1.3 log reduction at 2% and 4% against FL04, respectively. In contrast, MAS in the cotreatment with influenza viruses exhibited no antiviral effect (Figure 3(b)). Oseltamivir, a positive control, showed no inhibitory effects in the pre- and cotreatment against these viruses.

**Figure 4:**
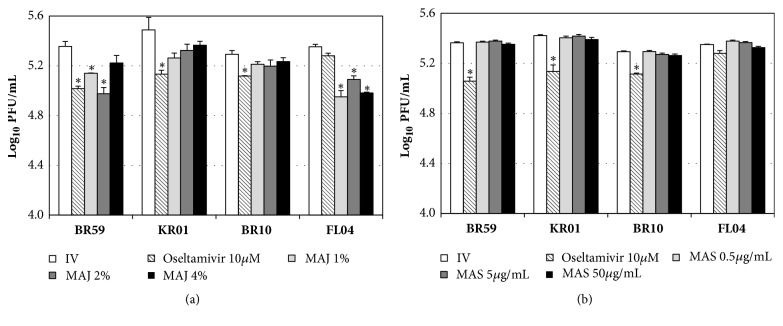
Antiviral activities of MAJ and MAS against BR59, KR01, BR10, and FL04 in posttreatment. After virus absorption to cells, the inocula were aspirated; then the cells were incubated with (a) MAJ and (b) MAS for 1 h. After incubation, the cells were washed and overlaid with agarose at 37°C for 72 h. Nontreated influenza virus (IV) and oseltamivir (10 *μ*M) were used as negative and positive controls, respectively. All measurements were performed in triplicate. Within each treatment, an asterisk denotes a significant decrease in the plaque-forming unit (PFU) (log_10_PFU/mL) to nontreated influenza virus group (P < 0.05).

In the posttreatment, MAJ showed low antiviral activities against BR59, KR01, and BR10, reaching 0.1-0.2 log inhibition at 4% concentration, except for 0.4 log inhibition of FL04 (Figure 4(a)). Oseltamivir at 10* μ*M exhibited 0.1-0.4 log inhibition against BR59, KR01, BR10, and FL04. In the case of MAS, no inhibition was observed compared to untreated controls (Figure 4(b)). Therefore, our time-of-addition experiments revealed that the highest antiviral effects against influenza viruses were achieved when viruses were treated with MAJ prior to viral infection or when cells were treated simultaneously with virus and MAJ. MAJ showed consistently stronger antiviral effects than MAS, regardless of strain subtypes and types. These findings suggest that MAJ can affect influenza virus entry into cells possibly by blocking attachment of the virus to host cells, or by inhibiting internalization of the virus into cells.

### 3.2. Identification of Polyphenol Compounds of MAJ

Polyphenol compounds of MAJ were quantitatively analyzed using LC-MS. Cyanidin-3-rutinoside (C3R) was the most abundant polyphenol compound (28.9 mg/g), which is followed by rutin, cyanidin-3-glucoside (C3G), 3,4-dihydroxybenzoic acid, chlorogenic acid, gallic acid, caffeic acid, and* p*-coumaric acid in the range of 0.01-5.8 mg/g (Table 1). It was reported that the major polyphenol compounds of MAS were caffeic acid, 3,4-dihydroxybenzoic acid, rutin, and C3R in the range of 1.3-1.7 mg /g [28].

### 3.3. Antiviral Effects of Polyphenol Compounds of MAJ

**Figure 5:**
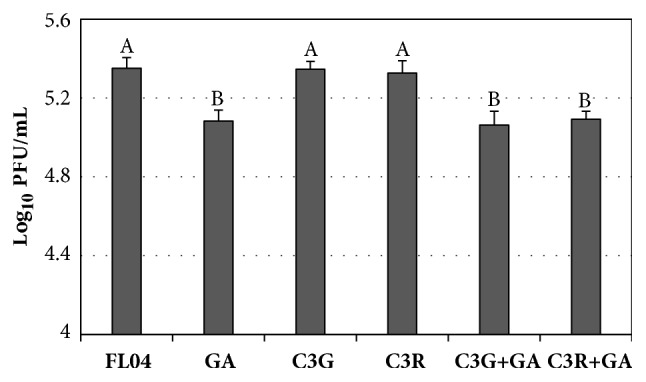
Antiviral activities of cyanidin-3-glucoside (C3G), cyanidin-3-rutinoside (C3R), and gallic acid (GA) from MAJ against FL04. The FL04 virus was mixed with polyphenol compound at 300 *μ*M for 1 h prior to virus infection. For the combination effect, the mixture of C3R at 300 *μ*M and GA at 300 *μ*M or the mixture of C3G at 300 *μ*M and GA at 300 *μ*M was tested. Nontreated influenza virus was used as a control. Reduction of plaque-forming unit (PFU) (log_10_PFU/mL) of influenza virus was evaluated using plaque assays. Different letters denote significant differences between treatments (P < 0.05).

MAJ showed strong antiviral effects in the pretreatment against FL04. The inhibitory effects of the single polyphenol compounds from MAJ were tested by pretreatment of FL04. After each polyphenol compound at a concentration of 300 *μ*M and FL04 were preincubated for 1 h at room temperature, MDCK cells were infected with the mixture. C3R and C3G showed little inhibitory effect, but gallic acid showed 0.2 log inhibition. The combinations of C3G and gallic acid and C3R and gallic acid showed no synergistic effects (0.2 log inhibitions) against FL04 (Figure 5).

### 3.4. Antioxidative Effects of MAJ

**Figure 6:**
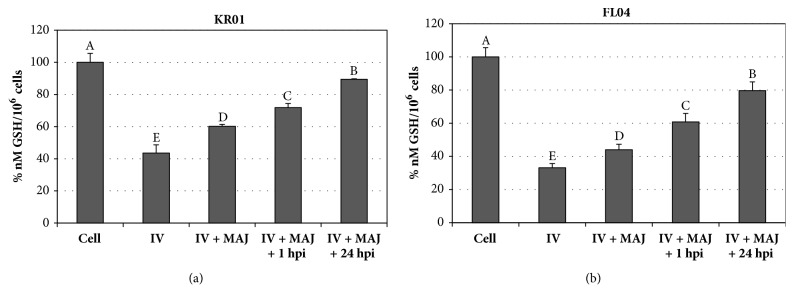
GSH levels in MDCK cell lysates. Cells were infected with (a) KR01 or (b) FL04, which were treated with 4% MAJ as follows: (1) nontreated influenza virus (IV), (2) virus-infected and cotreatment with MAJ (IV + MAJ), (3) cotreatment with MAJ and incubation 1 h after infection (IV + MAJ + 1 hpi), and (4) cotreatment with MAJ and incubation 24 h after infection (IV + MAJ + 24 hpi). The levels were expressed as percentage of nM/10^6^ cells (where levels from control cells were considered 100%). All measurements were performed in triplicate. Different letters denote significant differences between treatments (*P* < 0.05).

Since GSH exerts an essential buffering role against ROS, a deficiency of GSH in the cell can result in oxidative damage from ROS [30]. The KR01- or FL04-infected cells treated with 4% MAJ showed significantly increased GSH levels, compared to those in virus-infected cells (*P*<0.05): 17% and 11% increase in KR01 and FL04-infected cells, respectively (Figures 6(a) and 6(b)).

**Figure 7:**
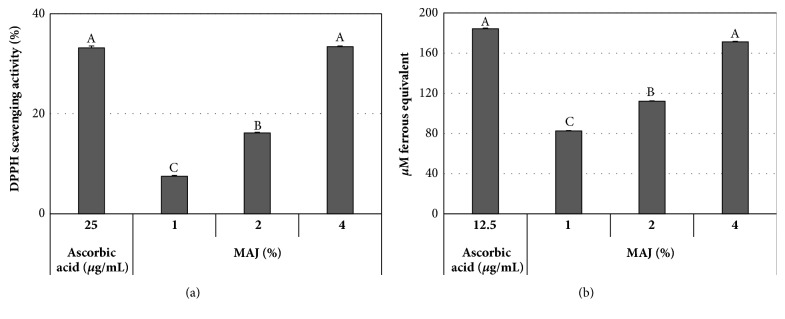
Antioxidant activities of MAJ. (a) DPPH radical scavenging activity and (b) FRAP assay results with the treatment of MAJ at different concentrations. Ascorbic acid was used as a positive control. All measurements were performed in triplicate. Different letters denote significant differences between treatments (*P* < 0.05).

Furthermore, MAJ showed significant effects as the scavenger of DPPH radical in a dose-dependent manner (Figure 7(a)). MAJ exhibited DPPH scavenging activities of 8-16% and 33% at concentrations of 1-2% and 4%, respectively. The latter activity by MAJ was equal to that of ascorbic acid at 25* μ*g/mL. In the FRAP assay, MAJ also exhibited ferric ion-reducing activity in a dose-dependent manner (Figure 7(b)). MAJ 4% showed 171* μ*M ferrous equivalent, corresponding to the effect of ascorbic acid at 12.5* μ*g/mL.

## 4. Discussion

Influenza virus infects cells in the upper respiratory tracts during the initial phase of infection, causing mild to severe illness. The virus is highly diverse with antigenic drift and shift, which can lead to the emergence of a virus that has not been exposed to the human population [40]. Recent severe influenza infections become a serious threat to children under the age of five, the elderly and chronic disease patients. Influenza B infections, which commonly strike later in the season, also impose a substantial public health burden, despite the existence of quadrivalent vaccines [41]. Although vaccines are available, there is still a great need for influenza antiviral drugs that reduce viral spread. There are numerous studies on the anti-influenza effects of natural products or plant-derived compounds. It was previously reported that pomegranate juice at 5% concentration decreased H3N2 virus titers by 5- to 10-fold [42] and guava tea at 4% concentration reduced influenza 2009pdm virus titers by 100% [11].* Adenium obesum* extract at 10 *μ*g/mL showed 99% inhibitory effect against H1N1 [19]. In the present studies, MAJ demonstrated significantly higher antiviral activities against the virus strains than MAS. The pretreatment of virus with MAJ prior to viral infection or the cotreatment exerted strong antiviral activity against influenza strains, BR59, KR01, and FL04 in a dose-dependent manner. Notably, the pre- and the cotreatment of MAJ at 4% inhibited FL04, an influenza B virus, with 1.3 log reduction, suggesting that MAJ had specific antiviral activity against FL04. In contrast, MAJ showed relatively lower inhibitory effects against BR10 (H3N2) than those against BR59 (H1N1) and KR01 (H1N1). Both H3N2 and H1N1 are influenza A viruses and belong to group 1 and 2 viruses, respectively, which are distant phylogenetically [43]. It was reported that patchouli alcohol showed little anti-influenza virus activity against A/Guizhou/54/89 (H3N2) but significant anti-influenza activity against A/PR/8/34 (H1N1) [44].

Given that the strong antiviral effect of MAJ was confirmed by viral plaque reduction assay, the mode of action of MAJ on influenza virus may be due to a direct inhibition at the initial stage of the attachment of the viral surface protein to its cellular receptor or internalization of virions into host cells. There are reports that plant extract resulted in inhibition of the attachment or internalization of influenza virus: cocoa extract and guava tea possess the same mechanisms in preventing virus adsorption to host cells [10, 11]. The inactivation of pomegranate polyphenol extract on influenza virus was mainly due to the damage of virion structural integrity, which was distinct from the effect on HA protein function [42] and associated with loss of hemagglutinating activity [45].

Influenza virus induces oxidative stress to activate redox-sensitive pathway useful for its viral replication. The redox-imbalance can be analyzed by an overproduction of ROS and a decrease of reduced GSH [30]. It has been reported that* Humulus lupulus* L. extract restored the glutathione content in the influenza virus-infected cells with radical scavenging and reducing activities, possibly due to the interference with redox-sensitive pathways required for viral replication [31]. In this study, KR01- or FL04-infected cells treated with 4% MAJ showed significantly increased GSH levels in the cotreatment for 1 h or 24 h. MAJ showed the significant DPPH radical and ferric ion-reducing activities in a dose-dependent manner, which contribute to counteracting the redox imbalance induced by viral infections. Taken together, our results suggest that the MAJ can interfere with the ROS-mediated cell damage caused by influenza virus infection.

Among polyphenol compounds of MAJ analyzed using LC-MS, C3R is the most abundant polyphenol compound, followed by rutin, C3G, 3,4-dihydroxybenzoic acid, chlorogenic acid, gallic acid, caffeic acid, and* p*-coumaric acid. It was reported that C3G and C3R were the main polyphenols of mulberry fruit [38]. Two major anthocyanins of* Morus alba* fruit were C3G and C3R [38]. The differences in composition and quantity of polyphenol compound of* Morus alba* depend not only on the species but also on the growing conditions, such as soil, geographical and environmental conditions during fruit development, the degree of maturity at harvest, and genetic differences. It was shown that single polyphenol compound revealed less significant antiviral activities against influenza viruses, despite strong antiviral effects of plant extract [45]; pomegranate polyphenol extract had anti-influenza activity, but ellagic acid, caffeic acid, and luteolin which are major polyphenol compounds of pomegranate did not show antiviral activities.* Hamamelis virginiana* bark extract showed higher antiviral activity against H1N1 influenza strains than any of its single polyphenol compounds such as gallic acid, tannic acid, and epigallocatechin gallate [46]. Quercetin and quercetin-3-glucoside showed a moderate antiviral effect, but other quercetin derivatives such as quercetin-3-galactose, quercetin-3-O-rutinoside, and quercetin-3-O-rhamnoside had minimal antiviral activity against influenza virus [47]. In this study, the pretreated virus with C3R or C3G prior to viral infection showed weak antiviral activity against FL04, whereas gallic acid, a minor polyphenol compound, revealed antiviral activity. When we tested antiviral effects of combinations of gallic acid and C3R or C3G in the pretreatment of virus, they showed no synergistic effect. It was shown that influenza viral replication was almost completely abolished by simultaneous treatment with a* Rubus coreanus* seed extract fraction at a concentration of 50 *μ*g/mL. Simultaneous treatment with gallic acid showed a concentration-dependent inhibition at relatively high concentration against influenza B virus [16]. Nevertheless, we cannot rule out the possibility that unidentified polyphenol compounds or other component in MAJ can contribute to its antiviral activity against influenza viruses.

MA fruit has a long history of use as an edible fruit and traditional medicine. A great diversity of bioactive compounds, including anthocyanins, rutin, quercetin, chlorogenic acid, and polysaccharides, have been found in MA fruit. Furthermore, MA fruit has shown numerous biological activities such as antioxidant, neuroprotective, antiatherosclerosis, immunomodulatory, antitumor, antihyperglycemic, and hypolipidemic activities [48]. Natural food products might be safe and ecofriendly without side effects. The significance of this study lies in the fact that mulberry, one of the popular berry fruits and juice consumed worldwide, can be utilized to control influenza viruses. In conclusion, the antiviral activity of MAJ against influenza strains was significantly higher than those of MAS against diverse subtypes and types of influenza viruses. MAJ at 2% and 4% exhibited 1.3 log inhibition on FL04 virus in the pretreatment and cotreatment of virus, respectively, and the significant enhancement of GSH levels in influenza virus-infected cells with strong antioxidant activity.

## Figures and Tables

**Table 1:** Contents of polyphenol compounds in MAJ using LC-MS.

Polyphenol compounds	Contents (mg/g dry weight)
Caffeic acid	0.05 ± 0.01
Catechin	ND
Chlorogenic acid	0.55 ± 0.01
*p*-Coumaric acid	0.01 ± 0.00
Cyanidin-3-glucoside	3.50 ± 0.05
Cyanidin-3-rutinoside	28.88 ± 0.03
3,4-Dihydroxybenzoic acid	0.74 ± 0.02
Ellagic acid	ND
Epigallocatechin-3-gallate	ND
*trans*-Ferulic acid	ND
Gallic acid	0.12 ± 0.01
Myricetin	ND
Quercetin	ND
Resveratrol	ND
Rutin	5.75 ± 0.17

Total	39.6

ND: Not detected.

## Data Availability

The data used to support the findings of this study are available from the corresponding author upon reasonable request.

## References

[1] Jennings L., Huang Q. S., Barr I. (2018). Literature review of the epidemiology of influenza B disease in 15 countries in the Asia-Pacific region. *Influenza and Other Respiratory Viruses*.

[2] Kadam R. U., Wilson I. A. (2018). A small-molecule fragment that emulates binding of receptor and broadly neutralizing antibodies to influenza A hemagglutinin. *Proceedings of the National Acadamy of Sciences of the United States of America*.

[3] Hause B. M., Collin E. A., Liu R. (2014). Characterization of a novel influenza virus in cattle and swine: proposal for a new genus in the Orthomyxoviridae family. *mBio*.

[4] Dou D., Revol R., Östbye H., Wang H., Daniels R. (2018). Influenza A virus cell entry, replication, virion assembly and movement. *Frontiers in Immunology*.

[5] Types of influenza viruses, https://www.cdc.gov/flu/about/viruses/types.htm

[6] Ison M. G. (2011). Antivirals and resistance: influenza virus. *Current Opinion in Virology*.

[7] Hsieh H.-P., Hsu J. T.-A. (2007). Strategies of development of antiviral agents directed against influenza virus replication. *Current Pharmaceutical Design*.

[8] Cheng P. K. C., To A. P. C., Leung T. W. C., Leung P. C. K., Lee C. W. C., Lim W. W. L. (2010). Oseltamivir- and amantadine-resistant influenza virus A (H1N1). *Emerging Infectious Diseases*.

[9] Wohlbold T. J., Krammer F. (2014). In the shadow of hemagglutinin: A growing interest in influenza viral neuraminidase and its role as a vaccine antigen. *Viruses*.

[10] Kamei M., Nishimura H., Takahashi T. (2016). Anti-influenza virus effects of cocoa. *Journal of the Science of Food and Agriculture*.

[11] Sriwilaijaroen N., Fukumoto S., Kumagai K. (2012). Antiviral effects of *Psidium guajava* Linn. (guava) tea on the growth of clinical isolated H1N1 viruses: Its role in viral hemagglutination and neuraminidase inhibition. *Antiviral Research*.

[12] Lee H. J., Lee Y. N., Youn H. N. (2011). Anti-influenza virus activity of green tea by-products in vitro and efficacy against influenza virus infection in chickens. *Poultry Science*.

[13] Theisen L. L., Muller C. P. (2012). EPs® 7630 (Umckaloabo®), an extract from *Pelargonium sidoides* roots, exerts anti-influenza virus activity in vitro and in vivo. *Antiviral Research*.

[14] Chavan R. D., Shinde P., Girkar K., Madage R., Chowdhary A. (2016). Assessment of anti-influenza activity and hemagglutination inhibition of *Plumbago indica* and *Allium sativum* extracts. *Pharmacognosy Research*.

[15] Kwon H.-J., Kim H.-H., Yoon S. Y. (2010). *In vitro* inhibitory activity of *Alpinia katsumadai* extracts against influenza virus infection and hemagglutination. *Virology Journal*.

[16] Lee J., Oh M., Seok J. (2016). Antiviral effects of black raspberry (*Rubus coreanus*) seed and its gallic acid against influenza virus infection. *Viruses*.

[17] Shoji M., Woo S., Masuda A. (2017). Anti-influenza virus activity of extracts from the stems of *Jatropha multifida* Linn. collected in Myanmar. *BMC Complementary and Alternative Medicine*.

[18] Thapa M., Kim Y., Desper J., Chang K.-O., Hua D. H. (2012). Synthesis and antiviral activity of substituted quercetins. *Bioorganic & Medicinal Chemistry Letters*.

[19] Kiyohara H., Ichino C., Kawamura Y. (2012). In vitro anti-influenza virus activity of a cardiotonic glycoside from *Adenium obesum* (Forssk.). *Phytomedicine*.

[20] Zu M., Yang F., Zhou W., Liu A., Du G., Zheng L. (2012). In vitro anti-influenza virus and anti-inflammatory activities of theaflavin derivatives. *Antiviral Research*.

[21] Ide K., Kawasaki Y., Kawakami K., Yamada H. (2016). Anti-influenza virus effects of catechins: A molecular and clinical review. *Current Medicinal Chemistry*.

[22] Palamara A. T., Nencioni L., Aquilano K. (2005). Inhibition of influenza A virus replication by resveratrol. *The Journal of Infectious Diseases*.

[23] DIng Y., Cao Z., Cao L., DIng G., Wang Z., Xiao W. (2017). Antiviral activity of chlorogenic acid against influenza A (H1N1/H3N2) virus and its inhibition of neuraminidase. *Scientific Reports*.

[24] Li R., Liu T., Liu M., Chen F., Liu S., Yang J. (2017). Anti-influenza A virus activity of dendrobine and its mechanism of action. *Journal of Agricultural and Food Chemistry*.

[25] Jacob J. R., Mansfield K., You J. E., Tennant B. C., Kim Y. H. (2007). Natural iminosugar derivatives of 1-deoxynojirimycin inhibit glycosylation of hepatitis viral envelope proteins. *Journal of Microbiology*.

[26] Gunjal S., Ankola A. V., Bhat K. (2015). In vitro antibacterial activity of ethanolic extract of *Morus alba* leaf against periodontal pathogens. *Indian Journal of Dental Research*.

[27] Bhuiyan M. I. H., Kim H.-B., Kim S. Y., Cho K.-O. (2011). The neuroprotective potential of cyanidin-3-glucoside fraction extracted from mulberry following oxygen-glucose deprivation. *Korean Journal of Physiology & Pharmacology*.

[28] Oh M., Bae S. Y., Chung M. S. (2013). Mulberry (*Morus alba*) seed extract and its polyphenol compounds for control of foodborne viral surrogates. *Journal of the Korean Society for Applied Biological Chemistry*.

[29] Lee J.-H., Bae S. Y., Oh M., Kim K. H., Chung M. S. (2014). Antiviral effects of mulberry (*Morus alba*) juice and its fractions on foodborne viral surrogates. *Foodborne Pathogens and Disease*.

[30] Amatore D., Sgarbanti R., Aquilano K. (2015). Influenza virus replication in lung epithelial cells depends on redox-sensitive pathways activated by NOX4-derived ROS. *Cellular Microbiology*.

[31] Di Sotto A., Checconi P., Celestino I. (2018). Antiviral and antioxidant activity of a hydroalcoholic extract from *Humuluslu pulus* L. *Oxidative Medicine and Cellular Longevity*.

[32] Fioravanti R., Celestino I., Costi R. (2012). Effects of polyphenol compounds on influenza A virus replication and definition of their mechanism of action. *Bioorganic & Medicinal Chemistry*.

[33] Bozzini T., Botta G., Delfino M. (2013). Tyrosinase and Layer-by-Layer supported tyrosinases in the synthesis of lipophilic catechols with antiinfluenza activity. *Bioorganic & Medicinal Chemistry*.

[34] Bizzarri B. M., Botta L., Capecchi E. (2017). Regioselective IBX-mediated synthesis of coumarin derivatives with antioxidant and anti-influenza activities. *Journal of Natural Products*.

[35] Rahman I., Kode A., Biswas S. K. (2007). Assay for quantitative determination of glutathione and glutathione disulfide levels using enzymatic recycling method. *Nature Protocols*.

[36] Shimada K., Fujikawa K., Yahara K., Nakamura T. (1992). Antioxidative properties of xanthan on the autoxidation of soybean oil in cyclodextrin emulsion. *Journal of Agricultural and Food Chemistry*.

[37] Benzie I. F. F., Strain J. J. (1996). The ferric reducing ability of plasma (FRAP) as a measure of 'antioxidant power': the FRAP assay. *Analytical Biochemistry*.

[38] Kim H., Kim S., Koh S. (2011). The development of natural pigment with mulberry fruit as a food additive. *Journal of Crop Science and Biotechnology*.

[39] Pawlowska A. M., Oleszek W., Braca A. (2008). Quali-quantitative analyses of flavonoids of *Morus nigra* L. and *Morus alba* L. (Moraceae) fruits. *Journal of Agricultural and Food Chemistry*.

[40] Medina R. A., García-Sastre A. (2011). Influenza A viruses: New research developments. *Nature Reviews Microbiology*.

[41] Reed C., Meltzer M. I., Finelli L., Fiore A. (2012). Public health impact of including two lineages of influenza B in a quadrivalent seasonal influenza vaccine. *Vaccine*.

[42] Sundararajan A., Ganapathy R., Huan L. (2010). Influenza virus variation in susceptibility to inactivation by pomegranate polyphenols is determined by envelope glycoproteins. *Antiviral Research*.

[43] Bissel S. J., Wang G., Carter D. M., Crevar C. J., Ross T. M., Wiley C. A. (2014). H1N1, but not H3N2, influenza a virus infection protects ferrets from H5N1 encephalitis. *Journal of Virology*.

[44] Kiyohara H., Ichino C., Kawamura Y., Nagai T., Sato N., Yamada H. (2012). Patchouli alcohol: *in vitro* direct anti-influenza virus sesquiterpene in *Pogostemon cablin* Benth. *Journal of Natural Medicines*.

[45] Haidari M., Ali M., Ward Casscells S., Madjid M. (2009). Pomegranate (*Punica granatum*) purified polyphenol extract inhibits influenza virus and has a synergistic effect with oseltamivir. *Phytomedicine*.

[46] Theisen L. L., Erdelmeier C. A., Spoden G. A. (2014). Tannins from *Hamamelis virginiana* bark extract: characterization and improvement of the antiviral efficacy against influenza A virus and human papillomavirus. *PLoS ONE*.

[47] Kim Y., Narayanan S., Chang K.-O. (2010). Inhibition of influenza virus replication by plant-derived isoquercetin. *Antiviral Research*.

[48] Yuan Q., Zhao L. (2017). The mulberry (*Morus alba* L.) fruit - a review of characteristic components and health benefits. *Journal of Agricultural and Food Chemistry*.

